# The relationship between bullism, depression and suicidal thought in adolescents in albania

**DOI:** 10.1192/j.eurpsy.2021.608

**Published:** 2021-08-13

**Authors:** F. Elezi, S. Tomori, K. Kaja

**Affiliations:** 1 Psychiatric Department, University Hospital Center “Mother Teresa”, Tirana, Albania; 2 Pediatric Department, University Hospital Center “Mother Teresa”, Tirana, Albania; 3 Emergency Department, Regional Hospital “Ihsan Cabej”, Lushnje, Albania

**Keywords:** Bullying, depression, suicidal thoughts, adolescents

## Abstract

**Introduction:**

The effects of bullying can be both physical and emotional, and they can last for many years. Children that experience verbal and physical bullying are at a greater risk of developing depression later on in life, compared with children who did not.

**Objectives:**

This study aims to look into the relationship between bullying, depressive symptomatology and suicidal thoughts in adolescents of high schools in Lushnje.

**Methods:**

Three questionaires (the Beck Depression Inventory; the Bully/victim Behavior / Victim Behavior Questionnaire by Olweus; the Suicide Questionnaire) were circulated online and were completed by 400 adolescents from 2 high schools in a small city in Albania between September-November 2019. Data has been analysed using the Software Package for Social Sciences for Windows v. 22.0 (SPSS Inc. Chicago, IL).

**Results:**

We found significant positive correlation between bullying (victimization) and the level of depression (r (n = 400) =. 300, p≤.05), and significant positive correlation of bullying (cause) with level of depression (r (n = 400) =. 160, p≤.05) but lower than in victimes. The victims of bullying have higher levels of depression and vice versa. We found higher rate of depression in female adolescents with the average (M = 14.710, ds = 11.263) compared to boys with the average (M = 9.609, ds = 10.723). There is an important positive correlation of suicidal ideation with the level of depression (r (n = 400) =. 616, p≤.05).

**Conclusions:**

Being either a bully or a victim of bullying seems to increase the chances of being affected by depression and suicidal thoughts

